# Profit and Pleasure

**Published:** 1871-01

**Authors:** Thos. K. Beecher


### THE BISTOURY.

VOL. VI. ELMIRA, N. Y., JAN., 1871. NO. 4.

### Communications.

### PROFIT AND PLEASURE.

BY THOS. K. BEECHER.

There is no place on earth so barren or so gloomy, that Nature (which is only another name for God) does not do something towards its ornamenting and beautifying. Moss grows even on gravestones. Ivy clings around the old ruin. The wind beautifies the edges of the snow drift that may have buried a score of travelers. The great wave that has just foundered a ship is daintily tipped with foam.

What beauty is to every square inch of nature, that, pleasure is to every, even the minutest act of duty. A certain ratio of pleasure is allotted to every well done duty. It is as sure to come as the moss and foam and drifted edges of which I spoke but now. But in either case it is very difficult to separate beauty from the place that produces it, and a very dangerous experiment to separate pleasure from the duty in which it has root. Boquets of flowers soon wither. Transplanted mosses grow rusty and seem out of place. And pleasures that are sought for, merely as pleasures very soon lose their high flavor and he that sought them his relish.

All which is an introduction to a little talk upon the various pleasures appointed to us through the gratification of appetite. To have strong appetites is a token of health. To be without them is one form of imbecility. Not to enjoy good food, or sleep, or the exhilaration of fresh air, or the gratification of the eye and ear, is a token not of piety and excellence, as the mistaken old ascetics imagined, but is evidence that one has missed the road that leads through manhood to immortality. Desire is the mainspring of life. But for hunger and thirst, the birds and beasts would hardly move, and nine tenths of the human race would sit supinely.

Every man is tempted, that is to say, he is tried or proved, when he has these strong desires. Power is power because it can explode. Steam is steam because it wants to get out. And man is man just in proportion to his elastic force, in other words his desires. A man is specially well off who finds himself endowed with strong appetites. His consciousness should be as of him who calmly drives a four-in-hand of mettlesome steeds. He has power enough for any emergency and laughs at danger *if his steeds are well bitted and under control.*

Danger and evil begin when men begin to be lovers of pleasure rather than lovers of God or good. Pleasure for pleasure's sake is always a wasteful and dangerous experiment—like a boy's squib, a waste of powder; and not like a miner's blast, which beside the delightful *bang*, heaves down three or four tons of coal besides—useful coal.

It is always dangerous to separate pleasure from the soil in which it was appointed to grow. Dangerous to force it or propagate it or multiply it by any artificial stimulants. It is because man having power to seek out many inventions is able thus to multiply pleasures, that man alone of all the beasts that God has created, is able to sin, because of the lust of the flesh and the lust of the eye.

As a railway engine after running thirty miles, tugging and panting, stops to wood and water, so a man that has worked has need of wood and water, that is, food and drink. No trick of cooking can ever devise such an appetizer or such a sauce as nature's own appetizer, good, honest, hard work.

Certain ancient gluttons had a way of feasting till they were full and then emptying themselves by a painless vomit; then they would feast again, as it were rinsing themselves with pleasures. Of course such pleasures are unnatural and short-lived. A man that minds his own business and does healthful work, will incidentally get all the pleasure that a man ought ever to get out of food and drink.

I suppose it to be uniformly and invariably a dangerous experiment to do anything for the mere pleasure of it. Here is where all drinkers become drunkards and tobacco users become slaves. Alcoholic drinks and tobacco and tea and coffee are all of them of highest use. By them we are able to satisfy the various conditions imposed upon us by modern civilization, and do full work and guard ourselves against evil consequences, by means of these and similar alteratives, appetizers and servants.

One man uses alcohol to stimulate him that he may go up in energy, and do more work of a certain kind for a little while than he otherwise could. Another man having gone up into the excitement of that work, uses alcohol to let him down. One has it as a ladder to climb by, the other as a ladder to come down by. The first is in danger, the second is safe.

"I don't see any harm in taking a little something now and then," says one. "I don't see any harm in dancing now and then," says another. "I don't see any harm in enjoying any pleasure that comes in my way so long as I don't swindle anybody."

There is always harm in any pleasure that is not first earned by aiming at some positive and definite good. I know of no exception to this rule. If we look for our duties, pleasures like flowers will grow up round our feet. If we go in chase of pleasure merely, we shall fill our bellies with east wind and come home with fists full of fog.

